# A208 ASSESSING FEASIBILITY AND ACCEPTABILITY OF AN ONLINE MIND-BODY WELLNESS PROGRAM FOR PRIMARY BILIARY CHOLANGITIS

**DOI:** 10.1093/jcag/gwab049.207

**Published:** 2022-02-21

**Authors:** M Watt, A Hyde, G M Wright, S Vander Well, J C Spence, A Mason, M McLeod, E Johnson

**Affiliations:** 1 Medicine/Gastroenterology, University of Alberta, Edmonton, AB, Canada; 2 University of Alberta, Edmonton, AB, Canada; 3 Medicine, University of Alberta, Edmonton, AB, Canada; 4 Canadian PBC Society, North York, ON, Canada; 6 Dalhousie University, Halifax, NS, Canada

## Abstract

**Background:**

Persons with primary biliary cholangitis (PBC) experience significantly higher rates of fatigue, stress, anxiety, depression, and impaired health related quality of life (HRQOL) as compared to the general population. While online wellness programming has been shown to be effective in decreasing fatigue and improving mental wellness in a variety of chronic disease populations, limited data is available for PBC.

**Aims:**

This pilot study aimed to assess the hypothesis that a 12-week, online, mind-body wellness program would be feasible (assessed through adherence and retention) and acceptable in people with PBC. We also aimed to explore indicators of impact on measures of wellbeing.

**Methods:**

Persons with PBC were recruited across Alberta and British Columbia in January 2021. The program included a 20–30 minute video containing low intensity mindful movement, meditation, and breathwork (goal 2–3 times/week) as well as a weekly behaviour change tip, PBC tip from a physician, and PBC nutrition tip. The online programming was accompanied by brief (10-minute) once weekly phone check-ins from a member of the study team, and optional once monthly zoom group sessions hosted by the Canadian PBC Society. Satisfaction and adherence were assessed at the end of the study using a survey. The pre-post exploratory efficacy assessment included: fatigue (Modified Fatigue Impact Scale), perceived stress (Perceived Stress Scale), anxiety and depression (Hospital Anxiety and Depression Scale), and HRQOL (PBC-40). Using a qualitative descriptive approach, we conducted semi-structured interviews at the end of the study to explore experiences with the intervention, and gather feedback for improvement.

**Results:**

Participants (N = 32) completed baseline surveys and 29 (91%) were retained to end-of-study. Twenty-five (86%) adhered to the program goal of carrying out the mind-body practice at least 2–3 days per week. Comparing baseline to end-of-study, significant reductions were observed in fatigue (13%, p=0.004), anxiety (30%, p=0.005), and depression (28%, p=0.022), and significant improvements were observed in the PBC-40 itch (22%, 0.043), fatigue (13%, 0.005), cognitive (17%, 0.006), and emotional (18%, 0.001) domains. Eleven individuals participated in qualitative interviews, reporting an increase in energy, a more positive outlook, and increased knowledge of PBC. Feedback supported acceptability (satisfaction score of 90%), with fatigue cited as the primary barrier to increased program participation.

**Conclusions:**

These findings suggest that a 12-week online mind-body intervention is feasible and acceptable to persons with PBC and has promising impact on efficacy. Recognizing the limitations of a single-arm study with a small sample size, a future RCT will be designed using this feedback.

**Funding Agencies:**

MITACS Accelerate, Canadian PBC Society

